# Clinical features and outcomes in primary nervous system histiocytic neoplasms

**DOI:** 10.1038/s41408-024-01083-x

**Published:** 2024-06-20

**Authors:** Nabeela Nathoo, Joon H. Uhm, Alyx B. Porter, Julie Hammack, Kurt A. Jaeckle, Maciej M. Mrugala, Brian A. Crum, Eoin P. Flanagan, Sean J. Pittock, Gaurav Goyal, Jason R. Young, Matthew J. Koster, Robert Vassallo, Jay H. Ryu, Caroline J. Davidge-Pitts, Corrie Bach, Aishwarya Ravindran, Julio C. Sartori Valinotti, N. Nora Bennani, Jithma P. Abeykoon, Mithun V. Shah, C. Christopher Hook, Karen L. Rech, Ronald S. Go, W. Oliver Tobin, Mayo Clinic-University of Alabama at Birmingham Histiocytosis Working Group, Lucinda M. Gruber

**Affiliations:** 1 Department of Neurology, Mayo Clinic, Rochester, MN USA; 2 Department of Neurology, Mayo Clinic, Phoenix, AZ USA; 3 Department of Neurology, Mayo Clinic, Jacksonville, FL USA; 4 Center for Multiple Sclerosis and Autoimmune Neurology, Mayo Clinic, Rochester, MN USA; 5 Department of Laboratory Medicine and Pathology, Mayo Clinic, Rochester, MN USA; 6 Division of Hematology-Oncology, University of Alabama at Birmingham, Birmingham, AL USA; 7 Department of Radiology, Mayo Clinic, Jacksonville, FL USA; 8 Division of Rheumatology, Mayo Clinic, Rochester, MN USA; 9 Division of Pulmonary and Critical Care Medicine, Mayo Clinic, Rochester, MN USA; 10 Division of Endocrinology, Mayo Clinic, Rochester, MN USA; 11 Division of Radiology, Mayo Clinic, Rochester, MN USA; 12 Division of Laboratory Medicine- Hematopathology section, Department of Pathology, The University of Alabama at Birmingham, Birmingham, AL USA; 13 Department of Dermatology, Mayo Clinic, Rochester, MN USA; 14 Division of Hematology, Mayo Clinic, Rochester, MN USA; 15 Division of Hematopathology, Department of Laboratory Medicine and Pathology, Mayo Clinic, Rochester, MN USA

**Keywords:** Haematological cancer, Neurological disorders, Signs and symptoms

## Introduction

Histiocytic neoplasms are characterized by abnormal accumulation of histiocytes (tissue-resident macrophages/dendritic cells), with or without associated stromal fibrosis and/or polymorphous inflammatory infiltrate [1, 2]. The most common histiocytic neoplasms in adults include Langerhans cell histiocytosis (LCH), Erdheim-Chester disease (ECD), and Rosai-Dorfman disease (RDD).

We aimed to determine the frequency of isolated nervous system involvement at initial evaluation, to characterize the clinical manifestations, imaging features, and response to treatment, and to investigate the frequency at which those with initial isolated nervous system disease later developed systemic involvement.

## Subjects and Methods

### Patient identification

The electronic medical record system was used to identify patients diagnosed with a histiocytic neoplasm evaluated at Mayo Clinic (January 1, 1996–February 28, 2023). Inclusion criteria were: (1) diagnosis of a histiocytic neoplasm based on nervous system tissue biopsy and appropriate clinical phenotype; (2) isolated nervous system involvement (unifocal, multifocal) without other organ system involvement based on initial body positron emission tomography (PET)/computed tomography (CT) or CT of the chest, abdomen, pelvis along with nuclear bone scan, if available; and (3) sufficient medical records for review. Cases that initially presented with isolated nervous system involvement and later developed systemic involvement were included. One previously published case was included [3].

Nervous system disease location was classified as dura, leptomeninges, supratentorial brain parenchyma, brainstem, cerebellum, pituitary, hypothalamus, spinal cord, and spinal nerve roots. Those with bone involvement (including calvarium and/or vertebrae) were excluded. Cases were reviewed by two neurologists (N.N and W.O.T).

### Clinical and laboratory data collection and review

Medical records were reviewed by a neurologist (N.N) for demographic, clinical, and laboratory data. *BRAF* V600E mutation testing results including the method of testing (tissue immunohistochemistry, tissue ddPCR, serum or urine cell-free DNA, and/or next-generation sequencing) were recorded.

Clinical response to therapy was classified based on prior reports [4, 5]: (1) complete response: complete resolution of signs and symptoms; (2) partial response: partial resolution of signs and symptoms; 3) stable disease: no change in signs and symptoms; 4) progressive disease: worsening of signs and symptoms, attributed to a histiocytic neoplasm. Similar to our previous study on ophthalmological manifestations of histiocytic neoplasm [6], the minimum duration considered for symptom improvement was 3 months [6]. Radiographic response to treatment was classified as above: (1) complete response, (2) partial response, (3) stable disease, and (4) progressive disease based on MRI brain and spinal cord findings.

### MRI review

Available MRIs of the brain and spinal cord were reviewed (N.N) for location of involvement and imaging features (enhancement, mass effect). Data was abstracted to a RedCap database.

## Results

### Frequency of isolated nervous system involvement at initial presentation in histiocytic neoplasms

Isolated nervous system involvement at initial presentation was seen in 19/377 (5%) adults with histiocytic neoplasms. This included 6/228 (3%) with LCH, 7/103 (7%) with ECD, and 6/46 (13%) with RDD.

### Demographics, clinical features, and laboratory characteristics

The median age at symptom onset was lowest in those with LCH (28.5 years, range 21–55) and highest in those with RDD (47.5 years, range 38–70) (Supplementary Table 1). There was a median delay of 7 months from symptom onset to final diagnosis, with the time to diagnosis being most delayed in LCH (13.5 months, range 1–60) and least delayed in RDD (7 months, range 1–20). The most common neurological symptoms and signs were headache (11/19, 58%), weakness (8/19, 42%), and diabetes insipidus (7/19, 37%) (Supplementary Table 2).

**Fig. 1:**
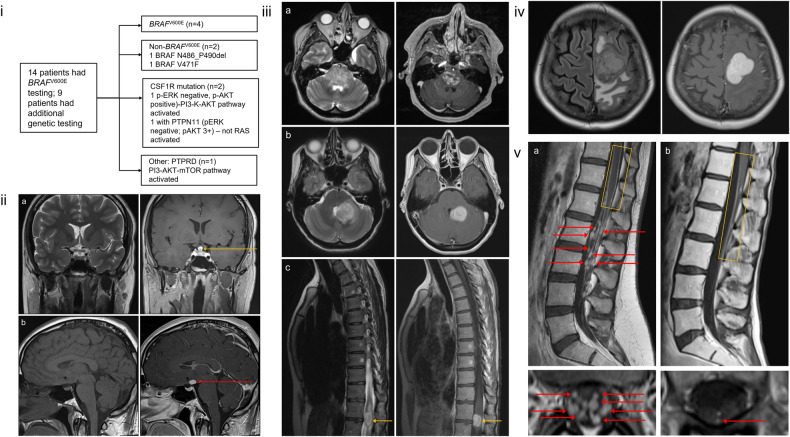
Genetic mutations and MRI findings in different types of primary histiocytic neoplasms. **i** Genetic mutations in patients with primary nervous system histiocytic neoplasms. The flow chart illustrates the number of patients in the series tested for genetic mutations and the associated pathways activated by these mutations. **ii** MRI findings in Langerhans cell histiocytosis (LCH). **a** Coronal brain MRIs of a man in his 20s with LCH presenting with diabetes insipidus. T2-weighted MRI (left image) and T1-weighted post-gadolinium MRI (right image), showing enhancement of infundibulum (yellow arrow). **b** Sagittal brain MRIs of a man in his 20s with LCH presenting with fatigue, weight gain, hypogonadism, and diabetes insipidus. T1-weighted pre-gadolinium MRI (left image) and T1-weighted post-gadolinium MRI (right image) demonstrate an ovoid heterogeneously enhancing lesion involving the hypothalamus (red arrow). **iii** MRI findings in Erdheim-Chester disease (ECD). **a** Axial brain MRIs of a woman in her 50s with ECD (*BRAF* N486_P490del). T2-weighted MRI (left image) and T1-weighted post-gadolinium MRI (right image) showing diffuse heterogenous enhancement of an enlarged pons. This was initially diagnosed as diffuse intrinsic pontine glioma. **b** Axial brain MRIs of a woman in her 30s with ECD (*BRAF* V600E+). T2-weighted MRI (left image) and T1-weighted post-gadolinium MRI (right image) shows an enhancing mass involving the left middle cerebellar peduncle and left cerebellar hemisphere with mass effect onto the 4th ventricle. **c** Sagittal spinal cord MRIs of a woman in her 30s with ECD (*BRAF* V600E+). T2-weighted MRI (left image) and T1-weighted post-gadolinium MRI (right image) show an enhancing intramedullary expansile mass at T12-L1 involving the conus (yellow arrows). **iv** MRI findings in Rosai-Dorfman disease (RDD). A woman in her 40s presented with progressive right leg weakness and imbalance and focal onset seizure with impaired awareness. Axial brain FLAIR MRI (left image) and T1-weighted post-gadolinium MRI (right image) demonstrate an extra-axial homogeneously enhancing mass with mass effect in the left frontal lobe. **v** MRI findings in ECD associated with CSF1R mutation. **a** Lumbar spine MRIs of a woman in her 20s presenting with progressive left leg weakness found to have ECD associated with CSF1R mutation. Sagittal lumbar spine T1-weighted post-gadolinium MRI (top left image) shows multiple enhancing nodules involving the cauda equina nerve roots (red arrows) with leptomeningeal enhancement of the lower thoracic cord and conus (yellow box). Axial lumbar spine T1-weighted post-gadolinium MRI (bottom left image) shows enhancing nodules (red arrows). **b** Lumbar spine MRIs of a woman in her 20s presenting with bilateral lower extremity numbness, gait imbalance, and neurogenic bladder and bowel, found to have ECD associated with CSF1R mutation. Sagittal lumbar spine T1-weighted post-gadolinium MRI (top right image) shows leptomeningeal enhancement of the lower thoracic cord and conus (yellow box). Axial lumbar spine T1-weighted post-gadolinium MRI (bottom right image) shows an enhancing cauda equina nerve root (red arrow).

*BRAF* V600E mutation testing was completed in 14/19 patients via tissue immunohistochemistry (11/14), tissue PCR (2/14), serum or urine cell-free DNA (6/14), and next-generation sequencing (4/14). Genetic testing results are summarized in Fig. 1i. Some samples were verified using multiple tests.

### Systemic imaging

All 19 patients underwent systemic imaging confirming the absence of systemic histiocytic neoplasm. F-18 fluorodeoxyglucose body PET/CT was undertaken in 16/19 patients. Tc99m methylene diphosphonate bone scan and CT of chest, abdomen, and pelvis were undertaken in 2/19 patients. Four of 19 patients had body PET/CT, nuclear bone scan, and CT of the chest, abdomen, and pelvis. One patient had a radiographic bone survey and CT of the chest.

The most recent time systemic imaging was completed after confirmation of nervous system involvement was a median of 30.7 months (range 0–225 months). In 3/19, systemic imaging was only completed at the time of CNS involvement. Sixteen of the 19 patients (84%) had sustained isolated nervous system involvement, with the latest median time of systemic imaging after nervous involvement being 7.5 months (range 0–225 months). Among the 3/19 patients (16%) who later developed systemic involvement, 2 had ECD with systemic involvement being detected at 5 months (bone involvement) and 11 months (widespread lymphadenopathy) after isolated nervous system involvement. One had LCH with systemic involvement detected 69 months (cutaneous involvement) after isolated nervous system involvement.

### MRI features

Abnormal enhancement of specifically affected regions was seen in all 19 patients (Supplementary Table 3). Thirteen of 19 (68%) had mass effect present.

In LCH, there was primarily pituitary (5/6) and hypothalamic involvement (4/6), seen as thickening and/or enhancement of the infundibulum (Fig. 1(ii)) or hypothalamus (Fig. 1(ii)). In ECD, the brainstem (5/7) (Fig. 1(iii)) and cerebellum (4/7) (Fig. 1(iii)) were primarily involved. An illustrative example of an ECD patient having an intramedullary expansile spinal cord mass is shown (Fig. 1(iii)). Four of 7 ECD patients had spinal nerve root disease; no patients with LCH or RDD had peripheral nervous system involvement. In RDD, isolated dural involvement was seen in the majority (5/6), which could present as a mass lesion (Fig. 1(iv)).

### Treatment and outcomes

Treatments undertaken included surgery, radiation, steroids, chemotherapy, other immunotherapy, BRAF inhibitors, and mitogen-activated protein kinase (MEK) inhibitors. Treatments used and clinical and radiologic responses are summarized (Table 1).

**Table 1:** Summary of Histiocytosis Involvement, BRAF V600E Mutation Status, Treatments Used, and Outcomes.

Case	Disease Type	Age at onset (decade range)	BRAF V600E mutation	Other mutations found	Initial Treatment(s)	Clinical response at 3 months	Radiologic response at 3 months	Subsequent Treatment(s)	Clinical response at last follow-up	Radiologic response at last follow-up
1	LCH	30–39	Not tested	Not tested	Radiation	SD	PR	None	SD	PR
2	LCH	20–29	−	Not tested	Surgery	SD	PR	None	SD	PR
3	LCH	20–29	Not tested	Not tested	Steroids	SD	PD	Surgery, cladribine	SD	PR
4	LCH	20–29	+	Not tested	Surgery	PR	CR	None	PR	CR
5	LCH	50–59	Not tested	Not tested	Cladribine	SD	SD	Radiation	SD	PR
6	LCH	30–39	−	Not tested	Radiation	SD	CR	None	SD	CR
7	ECD	30-39	+	Additional BRAF V471F mutation, NF1 splice site mutation and MCL1 amplification	Steroids, pegylated interferon alfa	PD	PD	Vemurafenib, radiation, cladribine, trametinib, binimetinib	PD	PD
8	ECD	50–59	−	BRAF p.N486/P490del	Steroids, plasma exchange, intravenous immunoglobulin	PD	SD	Rituximab, methotrexate, cobimetinib	PR	PR
9	ECD	40–49	+	Not tested	Steroids	N/A	N/A	Unknown	N/A	N/A
10	ECD	30–39	+	Not tested	Rituximab	PD	PD	Steroids, mycophenolate mofetil, vemurafenib	PR	PR
11*	ECD	18–19	−	CSF1R	Steroids, VP shunt, cobimetinib	SD	PD	Pegylated interferon alfa, methotrexate, binimetinib, pexidartinib, intra-arterial melphalan	PD	PD
12	ECD	20–29	−	PTPN11 E76K GOF, CSF1R	Steroids	PD	PD	Cobimetinib, cytarabine, pexidartinib, methotrexate	PD	PD
13	ECD	60–69	−	BRAF (D594A; c1781A>C, chromosome 7, position in 140453154)	Surgery, cobimetinib	SD	PD	N/A	SD	PD
14	RDD	30–39	Not tested	Not tested	Steroids	CR	SD	Azathioprine	CR	SD
15	RDD	40–49	−	No	Surgery, steroids	PD	PD	Cladribine, cytarabine, cobimetinib, clofarabine, proton beam therapy, lenalidomide	PD	PD
16	RDD	50–59	−	VUS in VHL and BRCA2 at levels suggestive of germline	Surgery, steroids	CR	PD	None	CR	PD
17	RDD	40–49	−	Not tested	Surgery	PR	CR	None	PR	CR
18	RDD	40–49	Not tested	Not tested	Surgery	CR	CR	None	CR	CR
19	RDD	70–79	−	VUS for UGT1A1, KMT2D, CYP3A5, VHL	Surgery, steroids	CR	PR	None	CR	PR

*CR* complete response, *ECD* Erdheim-Chester disease, *LCH* Langerhans cell histiocytosis, *N/A* not available, *PD* progressive disease, *PR* partial response, *RDD* Rosai-Dorfman disease, *SD* stable disease, *VUS* variants of uncertain significance; *previously published as a case report [3].

Among the 4 *BRAF* V600E-positive patients, 2 were treated with a BRAF inhibitor (vemurafenib). In 6/19 cases, an MEK inhibitor was used, which included cobimetinib, trametinib, or binimetinib. All 6 LCH cases had some response to treatment radiologically, but most remained with stable clinical disease given ongoing diabetes insipidus and endocrinopathies requiring replacement.

Eighteen patients had available clinical and radiologic follow-up data at 3 months and beyond. At the last clinical follow-up, 44% (8/18) had a response to treatment (complete, partial), 33% (6/18) had stable disease, and 22% (4/18) had progressive disease. At the last imaging follow-up, 61% (11/18) of patients had any response to treatment (complete, partial), 6% (1/18) had stable disease, and 33% (6/18) had progressive disease.

### *CSF1R* mutation-associated histiocytic neoplasms

Two ECD patients had a gain of function *CSF1R* mutation. They had a conserved clinical and MRI phenotype with leptomeningeal and lumbar nerve root involvement (Fig. 1(v)). They did not harbor any other myeloid neoplasm. Both were treated with pexidartinib. One patient had a partial response [3] for 3.5 years and then developed progressive disease; the other had a response for 1 year, but then developed progressive disease due to the development of malignant histiocytosis with *TP53* mutation.

## Discussion

Isolated nervous system involvement at the initial presentation of histiocytic neoplasms was seen in 5% of patients. The diagnosis was more delayed in LCH compared with RDD. RDD patients may be diagnosed faster due to the presence of dural-based lesions with mass effects resulting in seizure, headache, or weakness, leading to more rapid neuroimaging and biopsy/resection. A histiocytic neoplasm should be suspected in cases of diabetes insipidus, dural-based lesions, infiltrative brainstem lesions, and/or persistently enhancing lesions on brain or spinal cord imaging. An approach to the diagnosis of a potential histiocytic neoplasm has been published [7].

Most patients had isolated nervous system involvement even with systemic imaging completed after diagnosis. However, 2 ECD patients developed systemic involvement <12 months after presenting with isolated nervous system involvement. Thus, we would recommend that systemic imaging be completed for at least 12 months after nervous system involvement is confirmed. The LCH patient later developed cutaneous involvement not detected on systemic imaging. The threshold for biopsy in these situations should be low, even if it is several years after isolated nervous system involvement.

Responses to targeted therapy were neither universal nor complete. Only 2 patients were treated with BRAF inhibitors in our cohort, one with partial response to therapy and the other developing progressive disease. There was inconsistent response to MEK inhibitors, with 3 of 6 developing progressive disease. In cases of *CSF1R* gain of function mutation, we have noted a conserved clinical phenotype with leptomeningeal involvement with lumbar nerve root involvement. The two *CSF1R* cases in our cohort had primary CNS disease first, suggesting that the neoplastic clone may arise from a CNS resident embryonic precursor cell rather than a myeloid precursor cell.

In conclusion, we present a series of patients with primary CNS histiocytic neoplasms. This series raises the possibility of a progenitor cell line arising in the CNS, rather than in the systemic myeloid system. This entity should be considered in patients with persistent gadolinium-enhancing lesions in the brain or spinal cord. In patients with known primary CNS histiocytic neoplasms, systemic involvement can occur, underlining the need for long-term systemic surveillance imaging, in addition to monitoring CNS disease.

### Supplementary information

Supplementary Material

## Data Availability

Anonymized data not published within this article will be made available by request from any qualified investigator.
