# Supplementary material for: Clinical features and outcomes in primary nervous system histiocytic neoplasms
Source: Blood Cancer J. 2024 Jun 20;14(1):101. doi: 10.1038/s41408-024-01083-x (PMC11190138; doi:10.1038/s41408-024-01083-x)
Supplement: Supplementary file 1 — Supplementary Material [file 41408_2024_1083_MOESM1_ESM.docx]

**Supplementary Material**

**Supplementary Table 1. Demographic Features of Primary Nervous System Histiocytic Neoplasms**

| **Demographic Feature** | **All Subjects (n=19)** | **LCH (n=6)** | **ECD (n=7)** | **RDD (n=6)** |
| --- | --- | --- | --- | --- |
| Median age at symptom onset (range), years | 38 (19-70) | 28.5 (21-55) | 38 (19-65) | 47.5 (38-70) |
| Female sex (%) | 12 (63) | 2 (33) | 6 (86) | 4 (67) |
| Race/Ethnicity | 17 White, 1 Kuwaiti, 1 Puerto Rican | 6 White | 6 White, 1 Puerto Rican | 5 White, 1 Kuwaiti |
| Active Smoker (%) | 3 (16) | 0 (0) | 2 (29) | 1 (17) |
| Months follow-up, median (range) | 26 (0-264) | 48.5 (0-264) | 19 (3-92) | 19.5 (1-42) |
| Months follow-up from initial symptoms, median (range) | 42 (5-287) | 60.5 (11-287) | 56 (11-105) | 25.5 (5-54) |
| Weeks to symptom nadir, median (range) | 48 (0-420) | 34.5 (4-96) | 100 (0-420) | 13 (0-108) |
| Months from symptom onset to final diagnosis, median (range) | 7 (1-105) | 13.5 (1-60) | 12 (1-105) | 7 (1-20) |
| Alive at time of last follow-up (%) | 17 (89) | 6 (100) | 6 (86) | 5 (83) |

ECD: Erdheim-Chester disease, LCH: Langerhans cell histiocytosis, RDD: Rosai-Dorfman disease

| **Case** | **Disease Type** | **Age at onset (decade range)** | **Headache** | **Diabetes insipidus** | **Ataxia** | **Weakness** | **Paraesthesia** | **Cognitive impairment** | **Seizure** | **Vision impairment** | **Cranial neuropathy** |
| --- | --- | --- | --- | --- | --- | --- | --- | --- | --- | --- | --- |
| 1 | LCH | 30-39 | - | + | - | - | - | - | - | - | + |
| 2 | LCH | 20-29 | - | + | - | - | - | - | - | - | - |
| 3 | LCH | 20-29 | - | + | - | - | - | - | - | - | - |
| 4 | LCH | 20-29 | + | + | - | - | - | - | - | - | - |
| 5 | LCH | 50-59 | - | + | - | - | - | + | - | - | - |
| 6 | LCH | 30-39 | + | + | - | - | - | - | - | - | - |
| 7 | ECD | 30-39 | + | - | + | + | + | - | - | - | + |
| 8 | ECD | 50-59 | + | - | + | + | + | - | - | + | + |
| 9 | ECD | 40-49 | + | + | + | - | - | - | - | - | - |
| 10 | ECD | 30-39 | - | - | - | + | - | - | - | - | - |
| 11* | ECD | 18-19 | + | - | - | + | + | + | + | - | + |
| 12 | ECD | 20-29 | + | - | + | + | + | + | + | + | + |
| 13 | ECD | 60-69 | + | - | - | - | - | - | - | - | - |
| 14 | RDD | 30-39 | + | - | - | - | + | - | - | - | - |
| 15 | RDD | 40-49 | + | - | + | + | + | - | - | + | + |
| 16 | RDD | 50-59 | - | - | - | + | - | - | + | - | - |
| 17 | RDD | 40-49 | - | - | - | + | - | - | + | - | - |
| 18 | RDD | 40-49 | + | - | - | - | - | - | + | - | - |
| 19 | RDD | 70-79 | - | - | - | - | - | - | + | - | - |

**Supplementary Table 2. Neurologic Manifestations of Primary Nervous System Histiocytic Neoplasm**

ECD: Erdheim-Chester disease, LCH: Langerhans cell histiocytosis, RDD: Rosai-Dorfman disease; *previously published as a case report (3)

**Supplementary Table 3. MRI Findings in Primary Nervous System Histiocytic Neoplasms**

| **Case** | **Disease Type** | **Age at onset (decade range)** | **Dural involvement** | **Leptomeningeal involvement** | **Supratentorial Brain Parenchymal involvement** | **Pituitary involvement** | **Hypothalamic involvement** | **Brainstem involvement** | **Cerebellar involvement** | **Spinal cord involvement** | **Spinal nerve root involvement** | **Abnormal Enhancement** | **Mass Effect** |
| --- | --- | --- | --- | --- | --- | --- | --- | --- | --- | --- | --- | --- | --- |
| 1 | LCH | 30-39 | - | - | - | + | - | - | - | - | - | + | - |
| 2 | LCH | 20-29 | - | - | - | + | + | - | - | - | - | + | + |
| 3 | LCH | 20-29 | + | - | - | + | + | - | - | - | - | + | + |
| 4 | LCH | 20-29 | - | - | - | + | + | - | - | - | - | + | - |
| 5 | LCH | 50-59 | - | - | - | - | + | - | - | - | - | + | - |
| 6 | LCH | 30-39 | - | - | - | + | - | - | - | - | - | + | + |
| 7 | ECD | 30-39 | - | - | - | - | - | + | + | + | + | + | + |
| 8 | ECD | 50-59 | - | - | - | - | + | + | - | - | - | + | + |
| 9 | ECD | 40-49 | - | - | - | - | - | + | + | - | - | + | - |
| 10 | ECD | 30-39 | - | - | + | - | - | + | + | + | + | + | + |
| 11* | ECD | 18-19 | - | + | - | - | - | - | - | - | + | + | + |
| 12 | ECD | 20-29 | - | + | + | - | - | + | + | + | + | + | + |
| 13 | ECD | 60-69 | - | - | + | - | - | - | - | - | - | + | + |
| 14 | RDD | 30-39 | + | - | - | - | - | - | - | - | - | + | + |
| 15 | RDD | 40-49 | - | - | - | - | - | + | + | - | - | + | + |
| 16 | RDD | 50-59 | + | + | - | - | - | - | - | - | - | + | + |
| 17 | RDD | 40-49 | + | - | - | - | - | - | - | - | - | + | + |
| 18 | RDD | 40-49 | + | - | - | - | - | - | - | - | - | + | - |
| 19 | RDD | 70-79 | + | - | - | - | - | - | - | - | - | + | - |

ECD: Erdheim-Chester disease, LCH: Langerhans cell histiocytosis, RDD: Rosai-Dorfman disease; *previously published as a case report (3)
